# First description of an orthotopically located spontaneously ruptured non-seminomatous, metastatic germ cell tumor and review of literature

**DOI:** 10.1016/j.eucr.2026.103437

**Published:** 2026-04-07

**Authors:** Leonhard Buck, Katharina Vogt, Jakob Kohler, Pernilla Virginia Conrad, Konrad Hügelmann, Julian Risch, Dithild-Angelika Melchior, Severin Rodler, Jonas Jarczyk, Philipp Nuhn

**Affiliations:** a Department of Urology, University Hospital Schleswig-Holstein Campus Kiel, Kiel, Germany; b Department of General Surgery, Visceral, Thoracic, Transplant and Paediatric Surgery, University Hospital Schleswig-Holstein Campus Kiel, Kiel, Germany

**Keywords:** Testicular rupture, Non-seminomatous germ cell tumour, Yolk sac tumour, Acute scrotum, Inguinal orchiectomy, Scrotal hematoma

## Abstract

Spontaneous rupture of a testicular tumour is extremely rare. We report a 51-year-old man presenting with rapid, painless scrotal swelling without trauma. Imaging showed a ruptured left testicular mass with extensive hematoma, emergency exploration confirmed tumour perforation. Histology revealed a yolk sac-predominant non-seminomatous germ cell tumour. While initial staging was negative, early restaging before adjuvant therapy identified a new pulmonary metastasis. The patient received three cycles of BEP chemotherapy and achieved complete remission. He remains disease-free at two-year follow-up. This case emphasises prompt evaluation and early restaging in atypical acute scrotal presentations.

## 1 Introduction

Testicular rupture is an uncommon urological emergency, most often caused by blunt trauma to the scrotum.^1^^,^^2^ In rare cases, underlying testicular tumor can make the testicle susceptible to rupture even with minimal or no trauma.^3^^,^^4^ Only a few cases of spontaneous rupture of germ cell tumor have been reported, most of which involved non-metastatic or undescended testes. Atraumatic rupture of an orthotopic, metastatic, non-seminomatous germ cell tumor is exceptionally rare. In this article, we present a case of spontaneous tumor rupture in a previously asymptomatic patient, accompanied by rapidly progressive scrotal swelling. We also discuss the associated diagnostic challenges and implications for early oncological management.

## 2 Case report

A 51-year-old man with no prior medical history presented to our emergency department that evening with massive, painless swelling of his left testicle. The patient described the swelling as having slowly increased throughout the day.

Scrotal trauma was credibly absent. The patient stated that he had no abdominal or genital pain, no fever, no nausea, and no bladder emptying problems. Upon further inquiry, he stated that his left testicle had always been slightly larger than his right, but he had not noticed any hardening. The volume of the left testicle had remained constant.

**Fig. 1:**
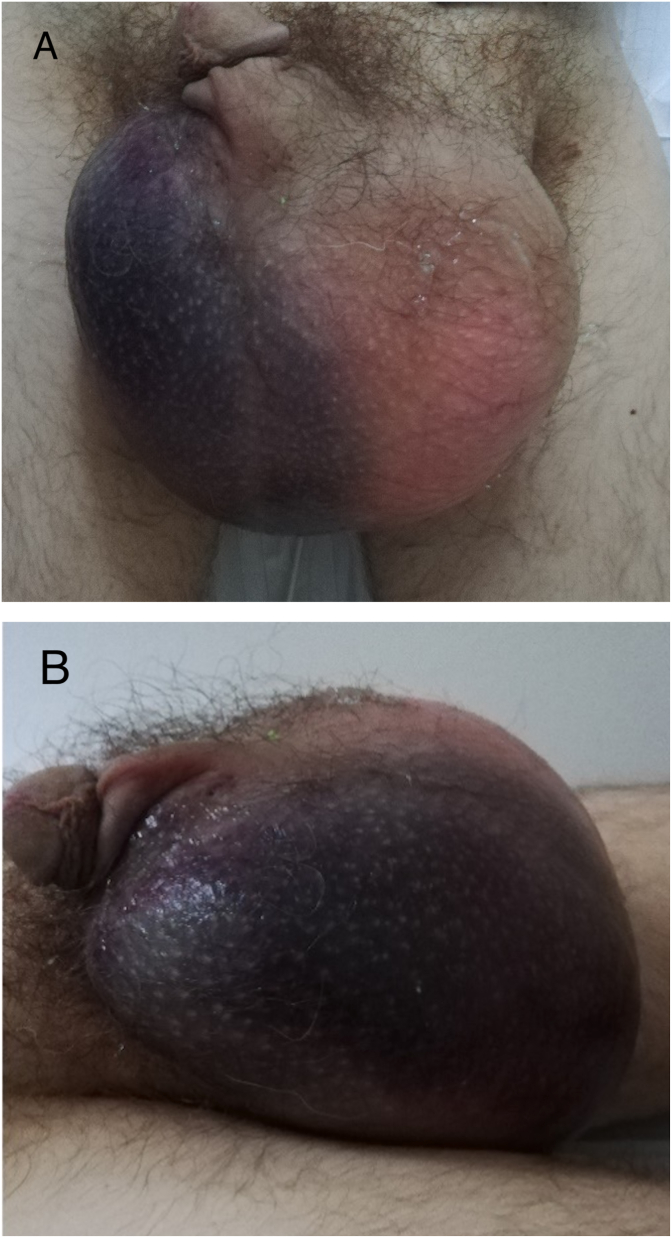
Massively swollen scrotum (A), with right-sided hematoma (B).

Physical examination revealed a massively swollen, handball-sized scrotum on the left side with an extensive hematoma of the right hemiscrotum and subsequent swelling of the left inguinal canal (Fig. 1A and B). The perineal raphe was distorted to the right.

Palpation revealed that the left hemiscrotum and the distal portion of the left inguinal canal were noticeably hardened. The narrower right hemiscrotum, in contrast, felt soft. The testes were not palpable on either side.

Laboratory examination revealed an elevated leukocyte count of 15x10^9/L, normal C-reactive protein, and an initial hemoglobin level of 10.2 g/dL.

**Fig. 2:**
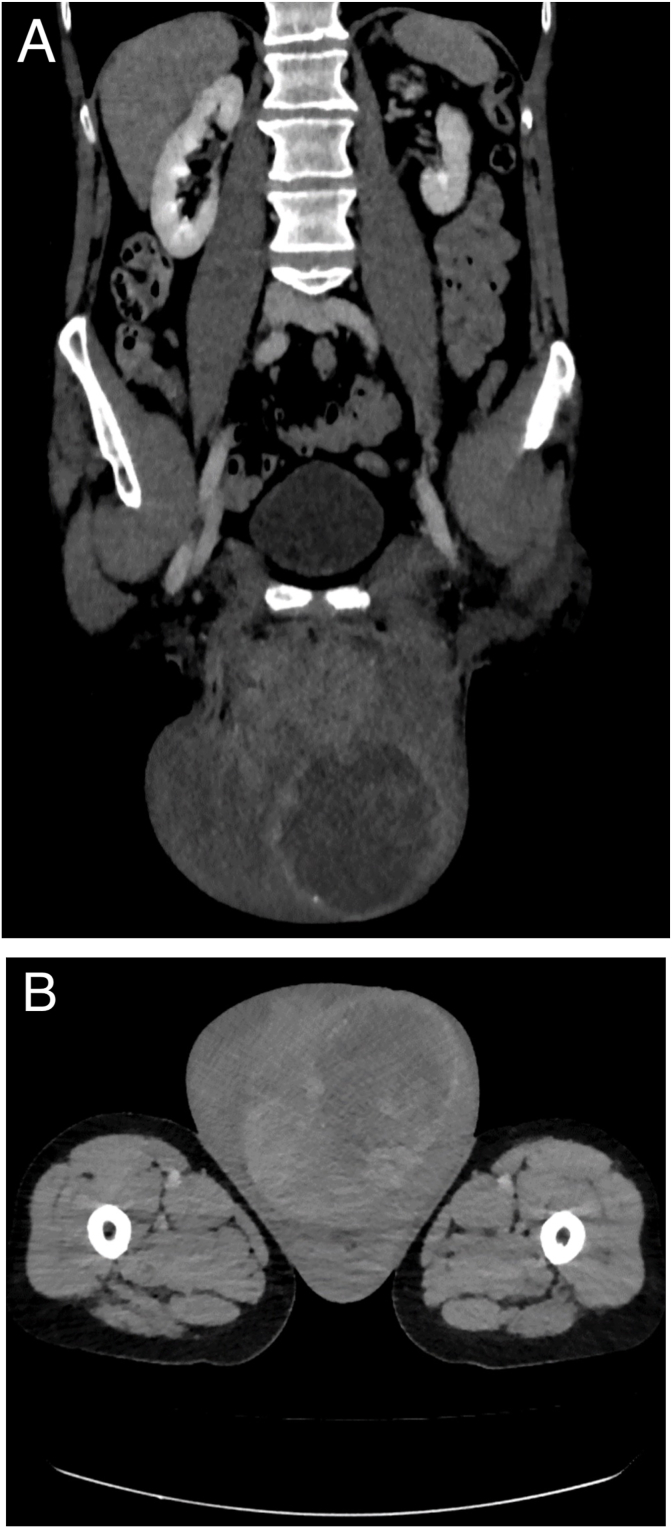
CT scan showing a ruptured scrotal tumour on the left side, in the coronal (A) and axial (B) planes.

Scrotal ultrasound showed a right-displaced, homogenous, and normally perfused right testicle. An unclear tissue mass was seen in the left scrotal compartment, some of which was clearly perfused and some of which was not. Additionally, small amounts of free fluid were found in the left scrotal compartment. Abdominal ultrasound was unremarkable. There was no evidence of an inguinal hernia. For further evaluation, we performed an emergency computed tomography (CT) scan of the abdomen and scrotum. CT scan confirmed the suspicion of a ruptured left testicular tumor. A pronounced hematoma was seen in the ipsilateral scrotal compartment, extending into the scrotal skin and the swollen left inguinal canal. The tunica albuginea appeared to be preserved in the left scrotal compartment as a hypervascularized ballooned structure. The preserved right testicle was displaced ventrolaterally to the right. No evidence of abdominal filiation was found (Fig. 2A and B).

At the time of the cross-sectional imaging, the patient had already been in the emergency room for approximately 6 hours. During this time, the volume of the scrotum continued to increase and was now tender to pressure. A blood gas analysis revealed a further decrease in hemoglobin of approximately 2 points to 8 mg/dL.

Therefore, an indication for emergency surgical exposure of the left testicle was established. Upon the patient's arrival in the operating room (8 hours after initial contact), the hemoglobin level was 6.3 mg/dL.

Tumor markers were obtained due to the suspicious imaging findings and showed elevated AFP levels of 15,000 μg/L (<15 μg/L), an elevated LDH of 547 U/L (60–120 U/L), and a normal HCG of <0.25 IU/L (<5 IU/L).

The left scrotal compartment was surgically exposed with an incision from the inguinal to the deep scrotal area (incision length approximately 20 cm). The inguinal dissection initially extended to the subcutaneous hematoma, which significantly hampered further dissection toward the inguinal canal. Upon reaching the inguinal canal, a high testicular detachment was performed. After further dissection toward the scrotum with circular exposure of the tunica albuginea, it was found to be completely perforated by tumor mass at the lower pole over an area of approximately 8 cm. All macroscopically visible tumor masses were removed, followed by extensive irrigation of the wound cavity and insertion of Easyflow® drains in the inguinal and scrotal areas.

The postoperative course was uneventful, with timely removal of the wound drains. During the inpatient follow-up, a CT scan of the thorax was added to the staging. This also revealed no abnormalities. The patient was discharged home after a few days.

Histopathology revealed a non-seminomatous germ cell tumor (>90% yolk sac tumor, <1% embryonal carcinoma) with extensive necrosis and capsular infiltration.

Tumor markers returned to normal values after approximately three months. The tumor stage according to the TNM classification was pT2 pNX V1 R0 with clinical stage IB according to the UICC.

**Fig. 3:**
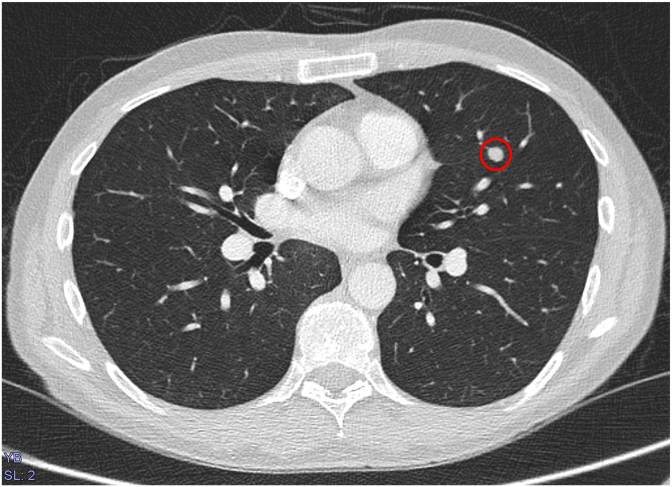
A newly developed metastasis in the left upper lobe.

The case was presented at the interdisciplinary tumor conference. The recommendation was to perform one cycle of polychemotherapy with bleomycin cisplatin, etoposide, and (BEP). Restaging was performed immediately before the start of chemotherapy. This revealed a newly developed pulmonary metastasis in the left upper lobe of the lung, prompting a stage upgrade to MIa according to UICC and three cycles of PEB (Fig. 3). The lung metastasis regressed after the first cycle. Two and a half years after completion of adjuvant chemotherapy, there is currently no evidence of recurrence (as of June 2025).

## 3 Discussion

Acute scrotum refers to the sudden onset of pain, redness, or swelling of the scrotum. The most common differential diagnoses are epididymitis/epididymo-orchitis, testicular torsion, and torsion of the testicular appendages. Various retrospective analyses show different frequencies for the individual entities.^5^^,^^6^

A testicular rupture is a relatively rare differential diagnosis in acute scrotum and constitutes an absolute urological emergency. Trauma is usually the cause of a testicular rupture, but in the case of testicular tumors, the tendency to rupture may also be increased even with minor trauma.^4^^,^^7^

Among the cases reported to date, there are also atraumatic ruptures of testicular tumors: Cutajar (1972) reported on the spontaneous rupture of a teratoma,^8^ LaMontagne (2002)^9^ reported on the atraumatic rupture of a non-seminomatous germ cell tumor of the scrotum. In this case, no metastasis was reported. To our knowledge, our case is the first atraumatically ruptured, metastatic non-seminomatous germ cell tumor. Spontaneous rupture of testicular tumors in cases of maldescensus has been described in several cases 10, 11, 12, 13. There are also reports of ruptures in non-urological tumors, for example in non-Hodgkin's lymphoma (NHL).^14^ Table 1 presents a literature review based on Luchey, Rogers, Saunder et al. (2014).^3^ To make the table clearer, cases with intraperitoneal rupture that were reported are not shown here.

**Table 1:** Ruptured testicular tumors reported in literature, based on Luchey, Rogers, Saunders et al. (2014)^1^.

Case	Author (Y)	Country	Age (years)	Trauma	Case	Treatment	Histology	Metastasis	Adjuvant Treatment	Follow-up
1^7^	Cassie (1956)	UK	22	Trivial 2 weeks previously	Testicular rupture	Scrotal exploration followed by inguinal orchidectomy	Seminoma	Lungs	Radiotherapy	NR
2^8^	Cutajar (1972)	UK	18	No	Testicular rupture	Inguinal orchidectomy	Teratoma	NR	Radiotherapy	18 months
3 ^15^	Liu (2001)	Taiwan	Neonate	No	Testicular rupture	Orchidectomy (not specified)	Mature cystic teratoma	NR	NR	8 months
4^9^	LaMontagne (2002)	USA	19	No	Testicular rupture	Inguinal orchidectomy	Mixed GCT, predominantly yolk sac	NR	3 cycles BEP	6 months
5^3^	Luchey (2014)	Canada	24	Crushed by tree	Testicular rupture	Inguinal orchidectomy	Mixed GCT	No	No	12 months
6^4^	Lunawat (2014)	UK	42	Kick in the scrotum	Testicular rupture	Scrotal orchidectomy	Seminoma	No	3 cycles BEP	12 months
7^16^	Asgari (2023)	Iran	23	NR	Testicular rupture	Inguinal orchidectomy	Mixed GCT	No	NR	NR

NR = not recorded; GCT = germ cell tumor

Ultrasound examination plays a crucial role in diagnosing testicular rupture. It reveals a rupture of the tunica albuginea with possible externalization of the testicular parenchyma. Doppler ultrasound can be used to detect reduced perfusion in the affected areas. Rapid surgical intervention within the first 72 hours enables a healing rate of 90%. After that, the rate drops to 45%.^17^ In such cases, further diagnostics can be performed using computed tomography. In particular, the risks and benefits of radiation exposure must be weighed up, especially in young patients.

If a testicular tumor is suspected, inguinal orchidectomy is the gold standard for surgical exploration. In emergency surgery and cases of rupture, en bloc resection can be significantly more difficult due to the rupture. Ruptures in cases of cryptorchism appear to carry an increased risk of peritoneal metastasis.^13^ In this case there was initially no indication of metastasis, but during an early restaging, a new lesion was detected in the lung. Disruption of the tunica albuginea with tumor tissue extrusion may facilitate the dissemination of malignant cells into the surrounding tissues or lymphovascular structures. Tumor spillage has been suggested as a potential risk factor for the early progression of the disease, particularly in aggressive non-seminomatous germ cell tumors.^18^ Early restaging therefore appears to be crucial, especially when tumor spillage due to rupture is suspected. If distant metastases are detected, chemotherapy with three to four cycles of BEP should be administered.^19^^,^^20^ This case highlights the importance of adjuvant chemotherapy in the metastatic stage of non-seminomatous germ cell tumors.

## 4 Conclusion

Spontaneous rupture of a testicular tumor is an extremely rare clinical event that requires a high index of suspicion, especially in the absence of trauma. In such cases, rapid diagnostic work-up and surgical intervention are critical, not only for symptom control, but also for histopathologic diagnosis and oncologic management. This case illustrates the need to consider underlying malignancy in atypical presentations of acute scrotal pathology and highlights the importance of interdisciplinary evaluation and timely initiation of adjuvant therapy to optimize patient outcomes.

## CRediT authorship contribution statement

**Leonhard Buck:** Writing – original draft. **Katharina Vogt:** Writing – review & editing. **Jakob Kohler:** Writing – original draft. **Pernilla Virginia Conrad:** Data curation. **Konrad Hügelmann:** Data curation. **Julian Risch:** Investigation. **Dithild-Angelika Melchior:** Methodology. **Severin Rodler:** Validation, Supervision. **Jonas Jarczyk:** Supervision. **Philipp Nuhn:** Supervision.

## Informed consent

Written informed consent was obtained from the patient for publication of this case report and accompanying images.

## Ethical approval

All data used in this study were derived from the patient's medical records. This report contains no personal information that could identify the patient; thus, ethical approval was waived.

## Funding

This research did not receive any specific grants from funding agencies in the public, commercial, or not-for-profit sectors.

## Declaration of competing interest

The authors declared no potential conflicts of interest with respect to the research, authorship, and/or publication of this article.
